# Detection and Potency Classification of Pre- and Pro-Haptens Using the SENS-IS Assay: The Role of Metabolically Competent Reconstructed Skin

**DOI:** 10.3390/toxics14080699

**Published:** 2026-08-07

**Authors:** Françoise Cottrez, Elodie Boitel, Essia Sahli, Marion Bastard, Christophe Capallere, Hervé Groux

**Affiliations:** ImmunoSearch Labs, Les Cyclades, 18 Chemin de Camperousse, 06130 Grasse, France; fcottrez@immunosearch.fr (F.C.); eboitel@immunosearch.fr (E.B.); esahli@immunosearch.fr (E.S.); mbastard@immunosearch.fr (M.B.); ccapallere@immunosearch.fr (C.C.)

**Keywords:** in vitro skin sensitization, SENS-IS assay, 3D reconstructed human epidermis (RHE), reconstructed in vitro full-thickness skin model (RhS), non-animal testing methods, pre- and pro-haptens

## Abstract

Skin sensitisation assessment increasingly relies on non-animal methods, yet the detection and characterisation of substances requiring metabolic activation, such as pre- and pro-haptens, remain challenging for several existing new approach methodologies (NAMs). The present study evaluated the performance of the SENS-IS assay using blinded testing of 50 substances reported in the literature as putative pre-haptens or pro-haptens on the historical EpiSkin™ model, in comparison with available LLNA, human and other NAM data. Additional experiments evaluated transferability to the current IS-SKIN model and the impact of a full-thickness skin model for selected metabolically demanding compounds. SENS-IS identified sensitisation as a hazard for the majority of substances in this pre- and pro-hapten-enriched dataset, including compounds requiring abiotic or metabolic activation, and provided potency-related categorisation broadly consistent with LLNA EC3 trends. Comparative analyses further supported comparable hazard identification and potency categorisation when SENS-IS was performed using the IS-SKIN and Episkin^TM^ reconstructed epidermis models for the tested subset of pro-haptens, supporting functional equivalence between tissues within the SENS-IS framework. For a limited subset of aromatic compounds reported to require extensive metabolic activation, sensitisation potency appeared to be underestimated in epidermal-only models. To address this limitation, the assay was applied to a full-thickness skin model. This approach improved potency characterisation of these metabolically complex compounds, while not increasing potency estimates for substances already adequately detected using epidermal models. Enhanced potency detection was associated with increased induction of phase I and phase II biotransformation genes included in the SENS-IS signature. Together, these results demonstrate that SENS-IS enables robust hazard identification and potency characterisation for a broad range of pre- and pro-haptens, and that the optional use of full-thickness skin models may extend its applicability to metabolically demanding substances while maintaining interpretability and avoiding non-specific signal amplification.

## 1. Introduction

Skin sensitisation is a major regulatory and public health concern, as it represents one of the most frequent causes of occupational and consumer-related adverse effects [1]. In consumer product sectors such as cosmetics and perfumery, where complex mixtures of chemically diverse ingredients are widely used, a substantial proportion of substances associated with skin sensitisation are known to require chemical or metabolic activation prior to exerting their sensitising potential [2].

It has been estimated that approximately 30–40% of fragrance allergens can be classified as pre-haptens or pro-haptens, relying on abiotic oxidation or enzymatic bioactivation to form the actual sensitising species [3,4]. Similar proportions have been reported across broader cosmetic ingredient inventories, highlighting the relevance of metabolic activation processes in the assessment of skin sensitisation within these industries. As a consequence, the reliable detection and characterisation of such substances represent a critical challenge for non-animal testing strategies [5].

Over the past decade, the replacement of animal testing by non-animal methods has led to the development of multiple new approach methodologies (NAMs) addressing individual key events (KEs) of the skin sensitisation adverse outcome pathway (AOP) [6]. While these methods have significantly improved hazard identification, limitations remain for the detection and potency characterisation of substances requiring chemical or metabolic activation, such as pre-haptens and pro-haptens [7].

Pre-haptens undergo abiotic transformation prior to skin exposure, whereas pro-haptens require enzymatic bioactivation within the skin to form reactive species capable of initiating the sensitisation process [4]. Several NAMs currently in use, including chemical reactivity assays and cell-based systems, have shown reduced sensitivity toward certain classes of pro-haptens, particularly those requiring complex metabolic activation [8]. To address this limitation, exogenous metabolic activation systems, such as S9 fractions, have been proposed. However, their relevance remains debated, as they may not accurately reflect skin-specific metabolism and can introduce non-physiological activation pathways [9,10].

The SENS-IS assay was developed as a genomic-based in vitro method designed to integrate multiple KEs of the skin sensitisation AOP within a single reconstructed human epidermis model [11]. By analysing the expression of a predefined gene signature encompassing pathways related to inflammation, oxidative stress, immune activation and biotransformation, SENS-IS enables simultaneous hazard identification and potency-related characterisation [12]. Previous studies have demonstrated that SENS-IS reliably detects both pre-haptens and pro-haptens without the need for exogenous metabolic activation systems, supporting the relevance of endogenous epidermal bioactivation [13].

Following the discontinuation of the EpiSkin™ reconstructed epidermis model [14], the SENS-IS assay was transferred to the IS-SKIN model. Ensuring biological and functional equivalence between epidermal models is essential for regulatory continuity, particularly for assays relying on endogenous metabolic competence.

Through our previous applications of the SENS-IS assay, we observed that the sensitisation potency of a limited subset of aromatic compounds requiring extensive metabolic activation—most notably polycyclic aromatic hydrocarbons—appeared to be underestimated when assessed using epidermal-only reconstructed skin models. Given the well-established complexity of the metabolic pathways involved for these substances, this observation was not interpreted as a limitation of the SENS-IS genomic approach itself, but rather as a consequence of the epidermal reconstructions, which may not fully recapitulate the complete biotransformation potential of human skin.

Based on this rationale, we hypothesised that improving the metabolic competence of the biological test system would allow a more accurate characterisation of sensitisation potency for such compounds, while preserving the robustness of hazard and potency assessment for substances already adequately detected using epidermal models. This reasoning led us to develop and evaluate a full-thickness skin model, incorporating both epidermal and dermal compartments, as an extension of the SENS-IS assay to test this hypothesis.

The present study aimed to (i) evaluate the performance of the SENS-IS assay for pre-haptens and pro-haptens in comparison with LLNA data, human reference data and other NAMs; (ii) demonstrate the equivalence of IS-SKIN and Episkin^TM^ reconstructed epidermis models within the SENS-IS framework; and (iii) investigate whether the use of a full-thickness skin model can extend potency characterisation for substances requiring extensive metabolic activation without leading to systematic overestimation of sensitisation potency. To minimize expectation bias and better reflect real-world industrial testing conditions, all substances were handled as coded samples and assessed under blinded conditions. Code breaking was performed only after predefined acceptance criteria were met and the primary analysis was finalized.

## 2. Materials and Methods

### 2.1. Reconstructed Human Skin Models

This study compared three in vitro human skin models: the IS-SKIN reconstructed epidermis model (ImmunoSearch, Grasse, France), the EpiSkin™ reconstructed human epidermis model (Episkin, Lyon, France), and a newly developed full-thickness (FT) skin model incorporating a dermal compartment. These models were used to evaluate the capacity of the SENS-IS assay to detect pre-haptens and pro-haptens.

#### 2.1.1. IS-SKIN Reconstructed Epidermis Model

The IS-SKIN model is a reconstructed human epidermis composed of primary human keratinocytes derived from foreskin and adult donors. Cells were cultured on polycarbonate inserts (surface area: 0.66 cm^2^) and differentiated under air–liquid interface conditions for 15 days to achieve epidermal stratification and functional barrier formation. The IS-SKIN model is routinely used in-house for SENS-IS applications and supports robust transcriptional responses across a broad range of test substances.

#### 2.1.2. EpiSkin™ Reconstructed Epidermis Model

The EpiSkin™ model was a commercially available reconstructed human epidermis composed of normal human keratinocytes cultured on a collagen matrix and maintained at the air–liquid interface (surface area: 1.02 cm^2^). EpiSkin™ has been extensively used as the reference tissue for the development and regulatory validation of the SENS-IS assay [11,12,13].

#### 2.1.3. Full-Thickness (FT) Skin Model

The full-thickness skin model consists of a dermal equivalent generated from type I bovine collagen populated with primary human dermal fibroblasts, overlaid with human keratinocytes from the same donor pool used for the IS-SKIN model. Following dermal gelation and contraction, keratinocytes were seeded onto the dermal matrix and cultured at the air–liquid interface for 17 days to allow complete epidermal stratification (surface area: 1.12 cm^2^). This model was developed to provide enhanced metabolic competence through the inclusion of a dermal compartment, while maintaining high tissue viability and epidermal differentiation.

**Figure 1:**
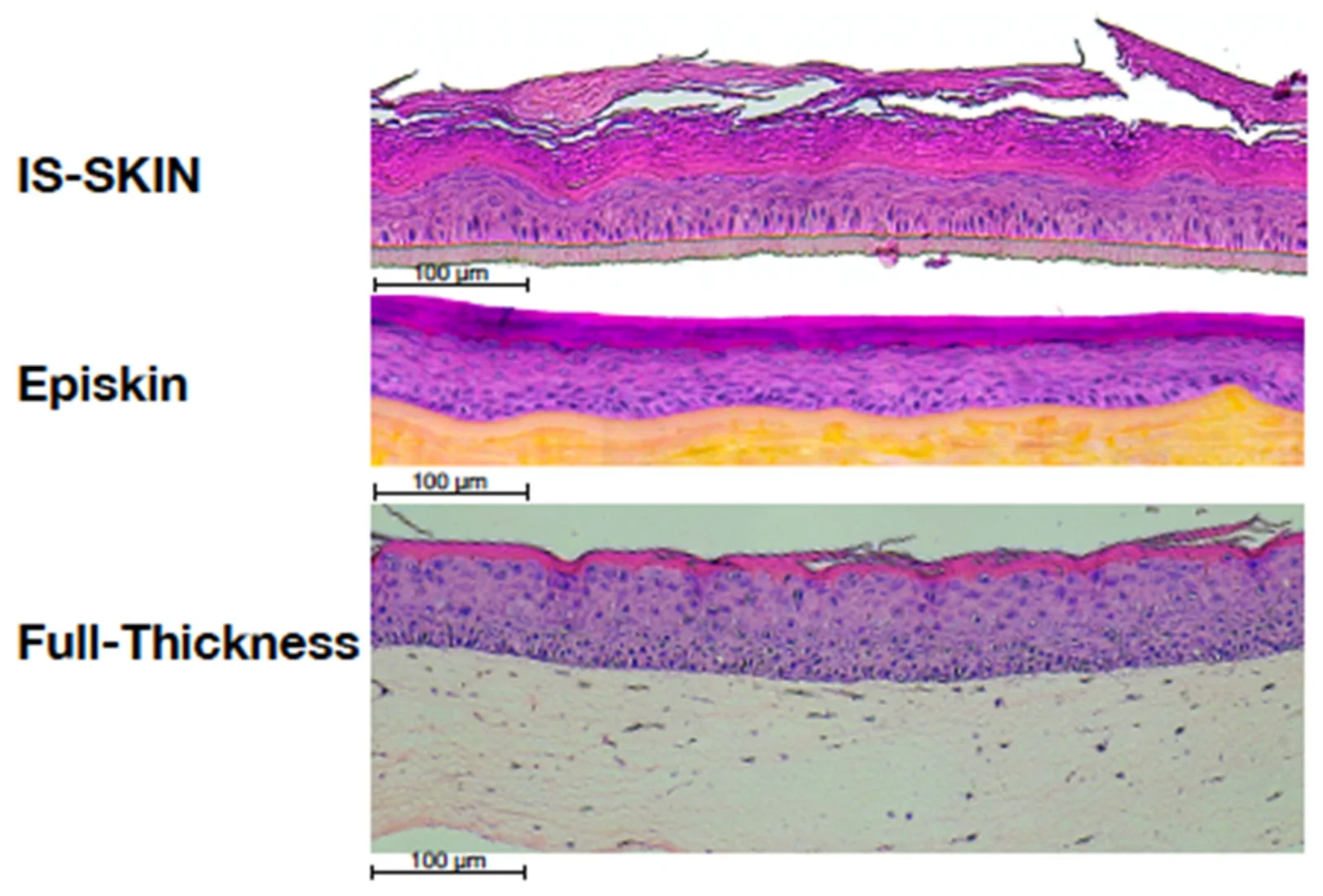
Representative histological appearance of reconstructed skin models used in this study. IS-SKIN-reconstructed human epidermis. EpiSkin™-reconstructed human epidermis. Full-thickness (FT) skin model incorporating both epidermal and dermal compartments. Sections were stained with hematoxylin and eosin (H&E). Scale bars: 100 µm.

Representative histological sections of the three reconstructed skin models are shown in Figure 1.

#### 2.1.4. Culture Conditions Prior to Exposure

IS-SKIN and full-thickness skin model inserts were maintained in 6-well plates with 1 mL of maintenance medium per well, whereas EpiSkin™ models were cultured in 12-well plates with 2 mL of medium per well. All tissues were incubated for 24 h at 37 °C, 5% CO_2_, and saturated humidity prior to substance exposure.

Additional technical details regarding tissue production, quality control, barrier function assessment and the standard implementation of the SENS-IS assay are described in detail elsewhere [11,14]. Prior to exposure, each reconstructed skin batch fulfilled the predefined SENS-IS tissue quality criteria, including histological evaluation, barrier function assessment and viability acceptance criteria, as previously described [11,14,15].

### 2.2. Test Substances

A total of 50 test items were evaluated, all selected based on reports from the literature describing them as putative pre-haptens or pro-haptens [8]. The test set included both reference substances and proprietary compounds, chosen for their reported reliance on abiotic or enzymatic activation and for their relevance to skin sensitisation assessment.

The full chemical panel is detailed in Table 1, including CAS numbers, EC3 values from LLNA (when available), known mechanistic activation pathways, and known human data. All chemicals were sourced at the highest purity available from commercial suppliers (Merck KGaA, Darmstadt; Germany, TCI Europe N.V., Zwijndrecht, Belgium, Bedoukian, Danbury, CT, USA).

Compounds were formulated in compatible solvents (e.g., PBS, DMSO, dipropylene glycol, and olive oil), with concentrations determined based on solubility. All substances were tested in a standard dose range (typically 50%, 10%, and 1% *w*/*v* or *v*/*v*), unless otherwise indicated.

All test items were handled as coded samples throughout the study to minimize expectation bias and to reflect routine industrial testing conditions. Coding was performed by personnel independent from the experimental team, and sample identities were not accessible to operators during tissue exposure, endpoint measurements, or data processing. All experimental procedures, quality control checks, and acceptance criteria were applied prior to unblinding. The primary statistical analysis outputs were finalized before code breaking (“analysis lock”). Test item identities were disclosed only after completion of quality control and confirmation of dataset integrity.

### 2.3. SENS-IS Assay and Prediction Model

Each formulation was topically applied to the tissue surface using a positive displacement pipette (30 µL for FT-skin and EpiskinTM; 19 µL for IS-SKIN). After 15 min at room temperature, excess material was removed with a 25 mL PBS rinse, and tissues were incubated for an additional 6 h at 37 °C, 5% CO_2_.

Each tested concentration was evaluated using two independent batches of reconstructed skin produced in separate manufacturing runs. When required according to the predefined decision tree, an additional independent batch was tested. Concordant results across independent tissue batches were required to validate the prediction, thereby controlling for batch-to-batch variability of the reconstructed epidermis.

RNA was extracted using Qiazol (Qiagen France SAS, Les Ulis, France) and the NucleoSpin 8 RNA kit (Macherey-Nagel SAS, Hoerdt, France), following manufacturer’s instructions. RNA quantity and quality were verified spectrophotometrically and via HSPA1A gene expression levels. Reverse transcription was performed using SuperScript III with random hexamers. Quantitative PCR (qPCR) was carried out using SYBR Green chemistry on the LC480 platform (Roche), targeting the predefined SENS-IS and Redox gene panels as previously described [16]. Normalization was done using the geometric mean of GUSB, B2M, and NONO reference genes.

The SENS-IS assay was performed according to the validated prediction procedure previously described [11,14,15]. Briefly, first, samples fulfilling the predefined cytotoxicity criteria were considered not interpretable for sensitization potency assessment and were excluded from potency interpretation, as previously described [16]. Substances were tested following the standard SENS-IS decision tree. Whenever technically feasible, testing was initiated at 10% (*w*/*v* or *v*/*v*) in two compatible vehicles. Depending on the outcome obtained at this initial concentration, additional concentrations (pure or 100% *w*/*v*, 50%, 10% or 1%) were evaluated according to the predefined decision tree until the lowest concentration fulfilling the SENS-IS positivity criteria was identified.

A tested concentration was considered positive when at least seven genes from either the SENS-IS or REDOX gene clusters were induced above the predefined threshold (1.25-fold versus the corresponding vehicle control) in both independent tissue batches. Potency was assigned according to the lowest concentration fulfilling these criteria: substances positive only when tested neat (or at 100% *w*/*v*) were classified as Very Weak, those positive at 50% were classified as Weak, those at 10% as Moderate, those at 1% as Strong and those at 0.1% as Extreme. Substances not fulfilling these criteria at any tested concentration were classified as non-sensitizers. 

Because SENS-IS is an algorithm-based prediction assay, data interpretation is based on predefined decision thresholds and concordance across independent reconstructed skin batches rather than on inferential statistical comparisons. Therefore, no hypothesis-testing statistical analyses were performed.

## 3. Results

### 3.1. Comparison of SENS-IS Outcomes with LLNA, Human Data, and Other NAMs for Pre- and Pro-Haptens

A total of 50 substances described in the literature as putative pre-haptens or pro-haptens were evaluated using the SENS-IS assay. SENS-IS results were compared with all available reference information for each substance, including LLNA outcomes, human evidence, and the principal validated in vitro NAMs (DPRA, KeratinoSens™ and h-CLAT), whenever such data were available (Table 1).

Based on LLNA data, the majority of substances included in the dataset were classified as sensitizers, with EC3 values spanning a wide range, from very low values indicative of high potency (e.g., ≤0.1) to high EC3 values associated with weak sensitizers. Human data were available for a subset of substances and were generally concordant with LLNA outcomes where reported.

The SENS-IS assay identified the large majority of LLNA-positive substances as sensitizers, including both pre-haptens and pro-haptens. This included substances requiring abiotic activation as well as compounds known to require metabolic activation. Overall, SENS-IS hazard calls (sensitizer/non-sensitizer) showed high concordance with LLNA classification, with a limited number of discordant outcomes.

In contrast, other NAMs showed variable detection of pro-haptens. While DPRA and KeratinoSens™ successfully identified several pre-haptens, a substantial proportion of pro-haptens yielded negative results in these assays. h-CLAT demonstrated intermediate performance, detecting some but not all pro-haptens included in the dataset.

### 3.2. Detection of Discordant Compounds Potentially Requiring Metabolic Activation

Two compounds, DEET and 6-methoxynaphthalene-2-carbaldehyde, are currently classified as non-sensitizers based on available LLNA data and lack convincing human evidence of skin sensitization. They were also reported as negative in the standard DPRA, KeratinoSens™, and h-CLAT assays performed in the absence of metabolic activation. However, both compounds were previously shown to become positive when KeratinoSens™ was performed in the presence of an S9 metabolic activation system [9].

In the present study, both compounds were reproducibly identified as weak sensitizers by the SENS-IS assay across independent reconstructed skin batches. In addition, a small number of case reports describing allergic contact dermatitis following DEET exposure have been published [17]. No comparable human evidence is currently available for 6-methoxynaphthalene-2-carbaldehyde.

### 3.3. Potency-Related Outcomes Provided by the SENS-IS Assay

In addition to binary hazard identification, the SENS-IS assay provided potency-related categorisation for sensitizing substances, ranging from Extreme to Weak. Substances associated with low LLNA EC3 values were generally assigned to higher potency categories (Extreme or Strong), whereas substances with higher EC3 values were predominantly classified as Moderate or Weak [16].

This potency stratification was observed across both pre-haptens and pro-haptens, including chemically diverse substances and compounds with different activation requirements.

### 3.4. Comparison of SENS-IS Outcomes Obtained with IS-SKIN and EpiSkin Reconstructed Epidermis Models

SENS-IS results obtained using the IS-SKIN reconstructed human epidermis model were compared with historical data generated using the EpiSkin™ model for a subset of 16 pro-haptens, all classified as sensitizers, including compounds used during the regulatory validation of the IS-SKIN model (Table 2).

For all substances included in the dataset, SENS-IS hazard calls obtained with IS-SKIN were concordant with those generated using EpiSkin™, with all pro-haptens consistently identified as sensitizers in both models.

Pro-haptens requiring epidermal bioactivation were detected with comparable efficiency using IS-SKIN [15] and EpiSkin™, with comparable hazard identification and potency categorization (Table 2).

Potency categorization was also consistent between the two models. Pro-haptens were assigned to the same or adjacent potency categories (Extreme, Strong, Moderate, or Weak), with no evidence of systematic over- or underestimation of potency observed with IS-SKIN compared with EpiSkin™ (Table 2).

Overall, comparable hazard identification and potency categorisation were obtained with IS-SKIN and EpiSkin™ for the tested set of chemicals.

### 3.5. Metabolic Gene Expression Profiles Following Exposure to Pre- and Pro-Haptens in Epidermal Models

**Figure 2:**
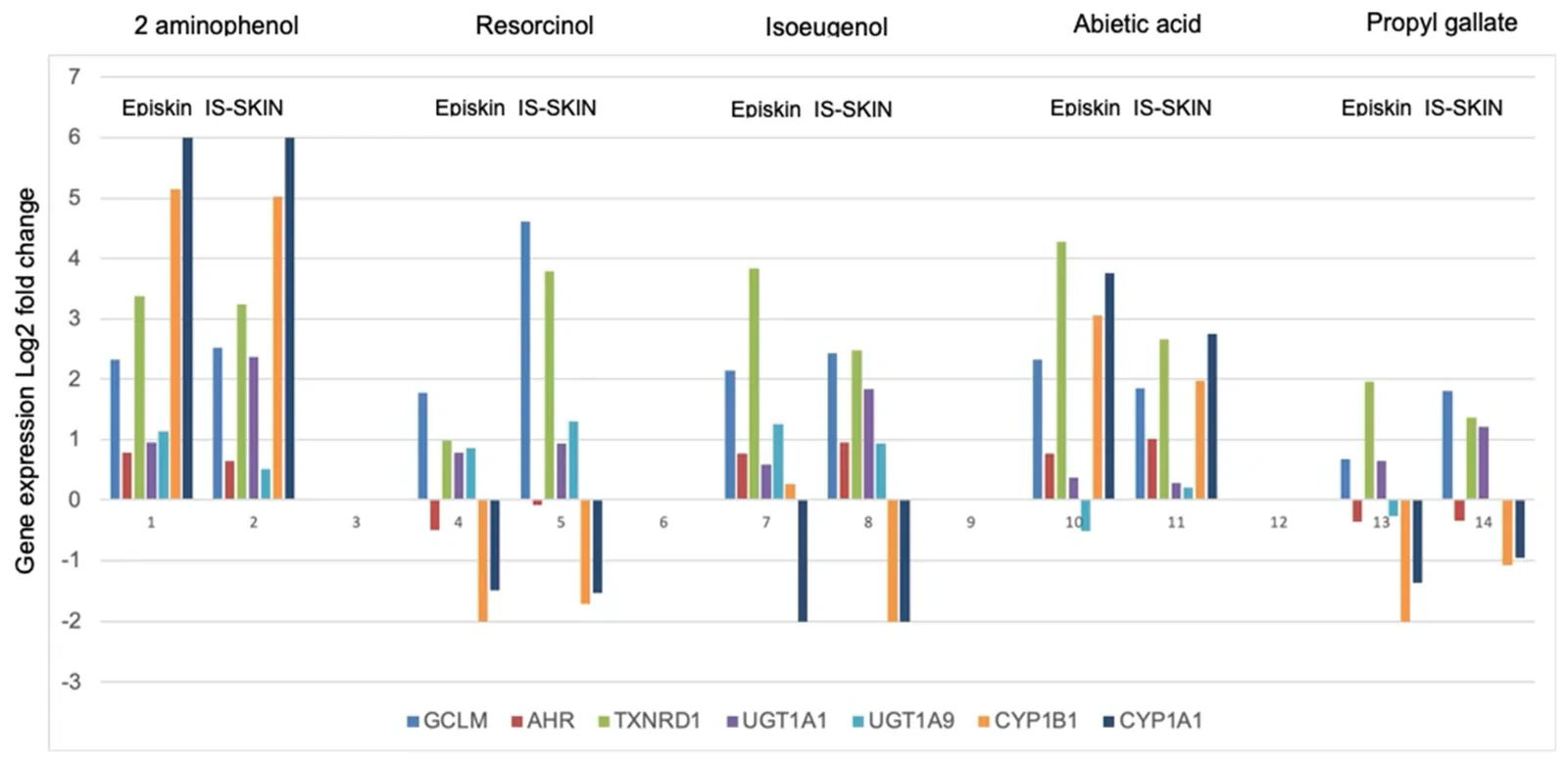
Expression of metabolism- and redox-related genes in response to pre- and pro-haptens in the SENS-IS assay. Gene expression changes for selected genes (GCLM, AHR, TXNRD1, UGT1A1, UGT1A9, CYP1B1 and CYP1A1) were measured following exposure to five pre- and pro-haptens (2-aminophenol, resorcinol, isoeugenol, abietic acid and propyl gallate). Results are expressed as log2 fold change relative to vehicle controls and were obtained in parallel using the EpiSkin™ and IS-SKIN reconstructed epidermis models. The gene expression profiles shown are representative of three independent SENS-IS determinations performed for each reconstructed epidermis model using independent tissue batches, all yielding concordant conclusions.

The expression of a selected panel of metabolism- and redox-associated genes (GCLM, AHR, TXNRD1, UGT1A1, UGT1A9, CYP1B1 and CYP1A1) was analysed following exposure to five pre- and pro-haptens (2-aminophenol, resorcinol, isoeugenol, abietic acid and propyl gallate) using the SENS-IS assay. Experiments were conducted in parallel with the EpiSkin™ and IS-SKIN reconstructed epidermis models (Figure 2).

Comparable gene expression profiles were obtained with both models across all tested substances, with no systematic differences observed between EpiSkin™ and IS-SKIN (Figure 2).

Substance-dependent patterns of metabolic gene modulation were observed. Exposure to 2-aminophenol and abietic acid was associated with increased expression of CYP-related genes, including CYP1A1 and CYP1B1, whereas resorcinol, isoeugenol and propyl gallate were associated with reduced expression of CYP genes. Modulation of phase II-related genes, such as UGT1A1 and UGT1A9, was observed for several substances (Figure 2).

### 3.6. Impact of Full-Thickness Skin Models on Potency Characterisation

**Figure 3:**
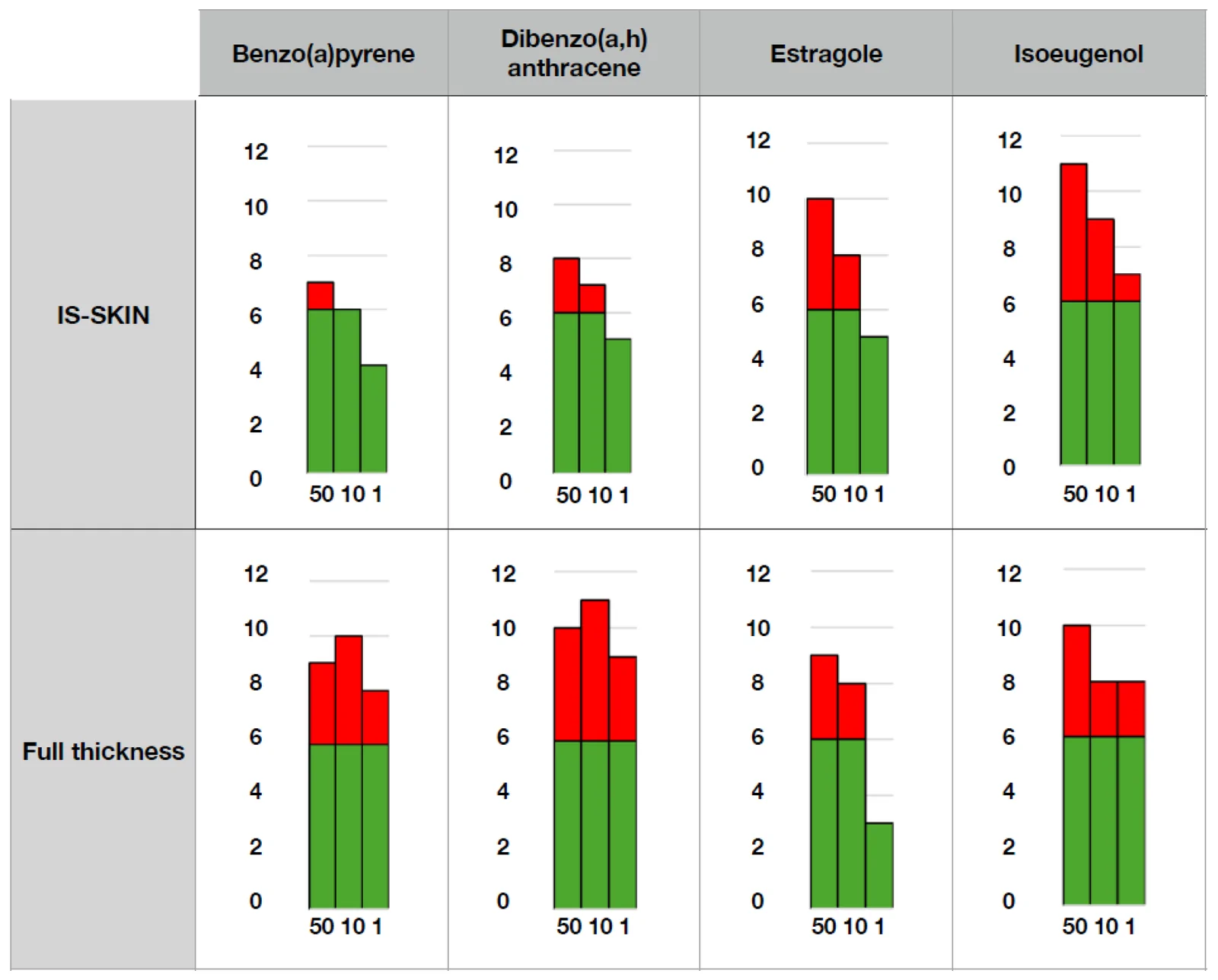
Impact of full-thickness skin models on potency assessment of selected aromatic compounds and pro-haptens. Green bars represent the number of positively responding genes within the SENS-IS and REDOX gene sets, whereas red bars indicate the number of additional genes required to reach the positivity threshold (7 genes). Reaching the positivity threshold at 50%, 10%, or 1% corresponds to a predicted weak, moderate, or strong sensitizer, respectively.

Through previous applications of the SENS-IS assay, we observed that among the 50 pre- and pro-haptens evaluated, only a limited subset of aromatic compounds requiring extensive metabolic activation—most notably benzo(a)pyrene and dibenzo(a,h)anthracene—appeared to have their sensitisation potency underestimated when assessed using epidermal-only reconstructed skin models (Table 1). Based on this observation, we hypothesised that this discrepancy could result from the limited metabolic competence of epidermal-only models. The present study therefore investigated whether the use of a full-thickness skin model could improve potency characterisation for these compounds. To challenge this hypothesis, estragole and isoeugenol, two metabolically activated compounds whose potency was already correctly predicted using epidermal models, were included as controls to determine whether the incorporation of a dermal compartment selectively improved potency assessment for metabolically demanding compounds without systematically increasing potency estimates (Figure 3). 

Skin sensitisation responses were assessed using the SENS-IS assay applied to an epidermal-only reconstructed skin model (IS-SKIN) and to a full-thickness skin model for four representative compounds: the polycyclic aromatic hydrocarbons benzo(a)pyrene and dibenzo(a,h)anthracene, and the pro-haptens estragole and isoeugenol. For each compound, responses obtained at three concentrations (50%, 10% and 1%) were compared between the two models in order to evaluate whether the inclusion of a dermal compartment could improve potency characterisation for substances requiring extensive metabolic activation. For each concentration, the bars represent the cumulative number of genes induced above the predefined SENS-IS expression threshold (1.25-fold versus the corresponding vehicle control). The green portion of the bar represents gene induction below the SENS-IS decision threshold (fewer than 7 induced genes), whereas the red portion represents additional induced genes beyond this threshold, indicating a positive SENS-IS prediction. Thus, bars extending into the red area correspond to sensitizing responses according to the SENS-IS prediction model.

When tested on the full-thickness skin model, benzo(a)pyrene and dibenzo(a,h)anthracene elicited stronger genomic responses at lower concentrations compared with those observed in IS-SKIN. This resulted in an apparent increase in sensitisation potency relative to the epidermal-only model. In contrast, when assessed using IS-SKIN, both compounds remained positive for sensitisation hazard but displayed weaker responses.

For estragole and isoeugenol, which are pro-haptens known to be adequately detected using epidermal models, comparable responses were observed in IS-SKIN and full-thickness skin models across the tested concentrations. In these cases, the inclusion of a dermal compartment did not result in an apparent increase in potency, and comparable responses were observed with both reconstructed skin models (Figure 3).

### 3.7. Relationship Between Enhanced Potency Detection and Biotransformation Gene Induction

**Figure 4:**
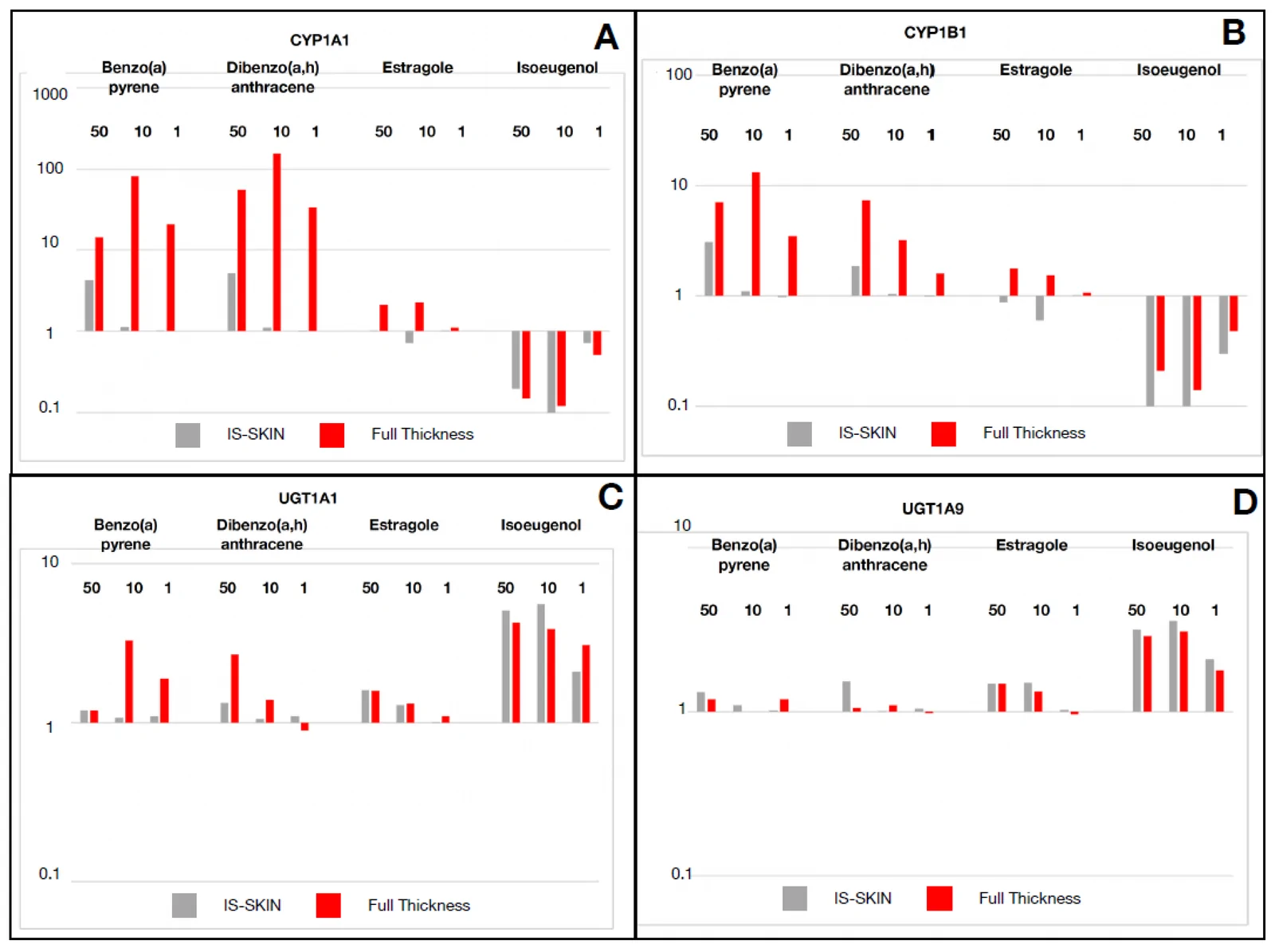
Comparative induction of SENS-IS signature biotransformation genes in IS-SKIN and full-thickness skin models. The expression of the phase I genes CYP1A1 (**A**) and CYP1B1 (**B**), and the phase II genes UGT1A1 (**C**) and UGT1A9 (**D**), which are part of the predefined SENS-IS genomic signature, was analysed following exposure of reconstructed skin models to four representative compounds (benzo(a)pyrene, dibenzo(a,h)anthracene, estragole and isoeugenol) at 50, 10 and 1%. Gene expression levels were determined in the epidermal-only IS-SKIN model and in the full-thickness skin model. Bars represent fold-change relative to the corresponding vehicle-treated controls and are displayed on a logarithmic scale. The profiles shown are one representative out of three independent SENS-IS determinations performed for each reconstructed skin model using independent reconstructed skin batches, all yielding concordant conclusions.

The comparative analysis of gene expression, focusing on selected biotransformation-related genes included in the SENS-IS signature, revealed marked differences between the epidermal-only IS-SKIN model and the full-thickness skin model (Figure 4).

For the phase I enzymes CYP1A1 and CYP1B1, exposure to benzo(a)pyrene and dibenzo(a,h)anthracene resulted in a pronounced, concentration-dependent induction in the full-thickness model. This response was consistently stronger than that observed in IS-SKIN, where induction remained moderate across all tested concentrations. In contrast, estragole and isoeugenol induced only limited changes in CYP1A1 and CYP1B1 expression in both models (Figure 4).

A similar pattern was observed for the phase II enzymes UGT1A1 and UGT1A9, which are also part of the SENS-IS transcriptional signature. The full-thickness model exhibited a robust upregulation of both UGT genes in response to benzo(a)pyrene and dibenzo(a,h)anthracene, whereas IS-SKIN showed weaker induction levels. For estragole and isoeugenol, UGT gene expression changes were modest and generally comparable between the two models.

Overall, stronger induction of CYP1A1, CYP1B1, UGT1A1 and UGT1A9 was observed in the full-thickness model for benzo(a)pyrene and dibenzo(a,h)anthracene, whereas comparable expression profiles were obtained for estragole and isoeugenol in both reconstructed skin models.

## 4. Discussion

The present study evaluated the performance of the SENS-IS assay using a dataset enriched in substances reported as pre- and pro-haptens, a particularly challenging class of chemicals for non-animal approaches because many require abiotic or metabolic activation before expressing their sensitizing potential. Across this dataset, SENS-IS showed a high level of concordance with available LLNA and human reference data for hazard identification while simultaneously providing potency-related categorisation broadly consistent with LLNA EC3 values, consistent with previous evaluations of the SENS-IS assay performed on broader datasets of sensitizing chemicals [16,18,19]. Comparable results were obtained following the transfer of the assay from the historical EpiSkin™ model to the IS-SKIN reconstructed epidermis, supporting the robustness and transferability of the SENS-IS approach [14]. As the dataset was intentionally enriched in pre- and pro-haptens, the present study was designed to evaluate the performance of the assay within this specific applicability domain rather than to estimate its overall sensitivity or specificity.

The present results nevertheless highlight an important aspect of quantitative skin sensitization assessment. While the reconstructed human epidermis provides sufficient endogenous metabolic activity to support hazard identification and potency categorisation for the large majority of pre- and pro-haptens included in this study, the results also indicate that this metabolic competence is not uniform across all chemical classes. Most compounds requiring abiotic or enzymatic activation were correctly identified and appropriately categorised using epidermal models alone, indicating that their metabolic requirements were adequately reproduced under these conditions [20,21,22]. However, a limited number of aromatic compounds requiring more extensive metabolic activation exhibited lower apparent potency than expected from the available reference data. Taken together, these observations indicate that, beyond the analytical performance of the transcriptomic assay itself, the biological capacity of the test system to reproduce the metabolic activation of test substances becomes an important determinant of quantitative potency assessment.

This hypothesis was investigated by comparing epidermal-only and full-thickness reconstructed skin models using four compounds selected to challenge the role of metabolic competence in potency assessment. Benzo(a)pyrene and dibenzo(a,h)anthracene were selected because they were the only compounds in the present dataset for which sensitisation potency appeared to be underestimated relative to the available reference data, whereas estragole and isoeugenol were included as controls since their potency was already appropriately characterised using reconstructed epidermis. For benzo(a)pyrene and dibenzo(a,h)anthracene, the full-thickness model produced stronger transcriptomic responses at lower concentrations together with increased induction of CYP1A1, CYP1B1, UGT1A1 and UGT1A9. In contrast, comparable transcriptomic responses and potency categorisation were obtained with both reconstructed skin models for estragole and isoeugenol. Taken together, these findings support the hypothesis that increasing the metabolic competence of the biological model, rather than modifying the transcriptomic assay itself, may improve potency characterisation for compounds requiring extensive metabolic activation.

The experimental observations obtained with the full-thickness model were accompanied by coordinated changes in the expression of genes involved in xenobiotic biotransformation. For benzo(a)pyrene and dibenzo(a,h)anthracene, the improved potency characterisation observed in the full-thickness model was associated with stronger induction of the phase I enzymes CYP1A1 and CYP1B1 together with the phase II enzymes UGT1A1 and UGT1A9. In contrast, comparable gene expression profiles were obtained for estragole and isoeugenol in both reconstructed skin models, consistent with the absence of any difference in potency categorisation.

Although enzyme activity or metabolite formation were not directly measured in the present study, previous work has demonstrated that induction of these genes in reconstructed human skin is associated with increased protein expression and enzymatic activity [23,24,25,26,27]. The coordinated modulation of phase I and phase II biotransformation pathways observed here therefore provides a biologically plausible explanation for the improved potency characterisation obtained with the full-thickness model, and is also consistent with the current understanding of cutaneous xenobiotic metabolism [23,24,25,26,27].

Taken together, these findings suggest that the improved quantitative performance is not an intrinsic property of the transcriptomic assay itself, but rather a consequence of applying the assay to a biological model with greater metabolic competence. This distinction is fundamental because it implies that the quality of quantitative skin sensitization assessment depends not only on the analytical method but also on the ability of the biological system to reproduce the metabolic activation required by the test substance.

The discordant observations obtained for DEET and 6-methoxynaphthalene-2-carbaldehyde further illustrate the importance of considering metabolic competence when interpreting sensitization data. Both compounds are currently classified as non-sensitizers based on available LLNA data and remain negative in conventional NAMs performed without metabolic activation. However, both were reproducibly identified as weak sensitizers by the SENS-IS assay and were previously reported to become positive in the KeratinoSens™ assay following S9-mediated metabolic activation. For DEET, isolated cases of allergic contact dermatitis have also been described in humans.

Although these observations are insufficient to redefine the sensitization status of these compounds, they highlight that the available evidence is not unequivocal. Rather than representing unequivocal false positives, these compounds may reflect situations in which differences in metabolic competence between experimental systems influence the final biological response. Such discordant compounds therefore represent valuable candidates for future mechanistic studies aimed at better understanding the contribution of cutaneous metabolism to skin sensitization assessment.

The present study has several limitations that should be considered when interpreting these findings. First, the evaluation of the full-thickness model was designed as a proof-of-concept evaluation to test a specific biological hypothesis rather than to provide a comprehensive validation across a large chemical dataset. Second, although the coordinated induction of biotransformation genes was consistent with previously established mechanisms of cutaneous metabolism, enzyme activity and metabolite formation were not directly measured in the present work. Finally, the discordant compounds identified in this study require further dedicated investigation before definitive conclusions regarding their sensitization status can be drawn.

These observations also provide practical guidance for the future application of reconstructed skin models. The choice of the biological model should be guided by the expected metabolic requirements of the test substance rather than by the analytical method itself. Reconstructed epidermis appears sufficient for the majority of sensitizers, including most pre- and pro-haptens evaluated in this study. In contrast, models with greater metabolic competence may provide additional value for compounds suspected to require extensive metabolic activation, for substances whose potency appears to be underestimated using epidermal models, and particularly for UVCBs, complex mixtures and finished products [28], where the individual contribution of each constituent to the overall sensitization response is often unknown and cannot be predicted a priori. In such situations, the use of a biologically more metabolically competent model may provide a more biologically relevant assessment of the overall sensitization potential of the product.

## 5. Conclusions

Overall, the present findings support a biology-driven strategy in which the physiological complexity of the biological model is adapted to the metabolic requirements of the test substance. Such an approach has the potential to improve quantitative skin sensitization assessment while preserving the mechanistic strengths of transcriptomic methods and supporting the continued evolution of human-relevant non-animal toxicology [28].

## Figures and Tables

**Table 1:** Comparison of skin sensitization hazard and potency outcomes for pre-haptens and pro-haptens obtained with the SENS-IS assay, the Local Lymph Node Assay (LLNA), human data, and other non-animal methods (NAMs).

Name	CAS	Type	LLNA	EC3	Human	DPRA	Keratinosens	hCLAT	SENS-IS S/NS	SENS-IS Potency
3 methylcatechol	488-17-5	pre-hapten	POS	0.02	NA	POS	POS	POS	POS	Extreme
1,4-phenylene diamine	106-50-3	pre-hapten	POS	0.16	POS	POS	POS	POS	POS	Strong
Hydroquinone	123-31-9	pre-hapten	POS	0.11	POS	POS	POS	POS	POS	Strong
2-nitro-1,4-phenylendiamine	5307-14-2	Pre- pro-hapten	POS	3.95	POS	POS	POS	POS	POS	Strong
2,5-diaminotoluene sulfate	615-50-9	pre-hapten	POS	0.4	POS	POS	POS	POS	POS	Strong
Metol	55-55-0	pre-hapten	POS	0.8	POS	POS	POS	NEG	POS	Strong
2-aminophenol	95-55-6	pro-hapten	POS	0.4	POS	POS	POS	POS	POS	Strong
Cinnamyl alcohol	104-54-1	pro-hapten	POS	21	POS	NEG	POS	POS	POS	Weak
5-amino-2-methylphenol	2835-95-2	pro-hapten	POS	1.92	POS	NEG	POS	POS	POS	Moderate
4-amino-m-creosol	2835-99-6	pre-hapten	POS	1.5	POS	POS	POS	POS	POS	Strong
4 allyl-anisol (Estragole)	140-67-0	pro-hapten	POS	20.20	POS	POS	NEG	POS	POS	Moderate
Eugenol	97-53-0	pro-hapten	POS	13	POS	NEG	NEG	POS	POS	Moderate
isoeugenol	97-54-1	pre-hapten	POS	1.2	POS	POS	POS	NEG	POS	Strong
4-chloroaniline	106-47-8	pre-hapten	POS	6.5	POS	NEG	POS	POS	POS	Moderate
Farnesol	4602-84-0	pro-hapten	POS	4.8	POS	NEG	POS	POS	POS	Moderate
Geraniol	106-24-1	pro-hapten	POS	26	POS	NEG	POS	POS	POS	Moderate
3-diethylamino propylamine	109-55-7	pro-hapten	POS	2.2	POS	NEG	POS	POS	POS	Moderate
Ethylenediamine	107-15-3	pro-hapten	POS	3.4	POS	NEG	POS	POS	POS	Moderate
Benzo(a)pyrene	50-32-8	pro-hapten	POS	0.22	POS	NEG	POS	POS	POS	Weak
Dibenzo(a,h)anthracene	53-70-3	pro-hapten	NA	NA	NA	NA	NA	NA	POS	Weak
Propyl gallate	121-79-9	pre-hapten	POS	0.32	POS	NEG	POS	POS	POS	Strong
Lauryl gallate	1166-52-5	pre-hapten	POS	0.3	POS	NEG	POS	POS	POS	Strong
Resorcinol	108-46-3	pre-hapten	POS	6.3	POS	NEG	NEG	POS	POS	Moderate
3-aminophenol	591-27-5	pro-hapten	POS	0.24	POS	NEG	NEG	POS	POS	Moderate
2-methoxy-4-methylphenol	93-51-6	pre-hapten	POS	5.6	POS	NEG	NEG	POS	POS	Moderate
Abietic acid	514-10-3	pre-hapten	POS	11.0	POS	NEG	POS	NEG	POS	Weak
Diethylenetriamine	111-40-0	pro-hapten	POS	3.3	POS	NEG	NEG	NEG	POS	Weak
N,N-dibutylaniline	121-69-7	pro-hapten	POS	NA	NA	NEG	NEG	NEG	POS	Moderate
Embramine hydrochloride	13977-28-1	pro-hapten	POS	5.5	NA	NA	NA	NA	POS	Moderate
Chlorothalonil	1897-45-6	pre-hapten	POS	0.004	NA	POS	POS	POS	POS	Extreme
Trifluralin	1582-09-8	pre-hapten	POS	5.8	NA	NA	NA	NA	POS	Moderate
Hexyl salicylate	6259-76-3	pre-hapten	POS	0.18	POS	NEG	NEG	POS	POS	Strong
1-Naphthol	90-15-3	pro-hapten	POS	1.3	NA	POS	POS	POS	POS	Moderate
Benzoyl peroxide	94-36-0	pre-hapten	POS	0.07	POS	POS	NEG	NEG	POS	Moderate
Bourgeonal	18127-01-0	pre-hapten	POS	4.3	POS	POS	NEG	POS	POS	Weak
Benzyl benzoate	120-51-4	pre-hapten	POS	17	POS	NEG	NEG	POS	POS	Weak
1,1,3-Triméthyl-3-phénylindane	3910-35-8	pre-hapten	POS	43	NA	NA	NA	NA	NEG	NS
Limonene	5989-27-5	pre-hapten	POS	52	POS	NEG	POS	NEG	POS	Weak
Citral	5392-40-5	pro-hapten	POS	4.1	POS	POS	POS	POS	POS	Moderate
Linalool	78-70-6	pro-hapten	POS	15	POS	NEG	POS	NEG	POS	Weak
Benzisothiazolinone	2634-33-5	pro-hapten	POS	4.8	POS	POS	POS	POS	POS	Moderate
Methylisoeugenol	6379-72-2	pro-hapten	POS	NA	NA	NA	POS	NA	POS	Weak
Creosol	93-51-6	pre-hapten	POS	5.6	POS	NEG	POS	NEG	POS	Moderate
Methyl eugenol	93-15-2	pro-hapten	POS	NA	POS	NA	POS	NA	POS	Weak
2-Ethoxy-4-methyphenol	203-184-2	pre-hapten	POS	NA	POS	NA	POS	NA	POS	Weak
Dihydroreugenol	2785-87-7	pro-hapten	POS	6.8	POS	NEG	NEG	POS	POS	Moderate
Carvone oxime	31198-76-2	pro-hapten	POS	NA	POS	NA	NEG	NA	POS	Weak
Aniline	62-53-3	pro-hapten	POS	89	POS	NEG	POS	NEG	POS	Weak
DEET	134-62-3	pro-hapten	NEG	NA	NEG	NEG	NEG/ POS (S9 act)	NEG	POS	Weak
6-Methoxynaphthalene-2-carbaldehyde	3453-33-6	pro-hapten	NEG	NA	NEG	NEG	NEG/ POS (S9 act)	NEG	POS	Weak
Sensitivity (%)			0.96		0.95	0.37	0.66	0.70	0.98	

The table summarizes skin sensitization results for a panel of substances classified as pre-haptens, pro-haptens, or metabolically activated pro-haptens (Pro-hapten (S9 act)), tested using the SENS-IS assay and compared with available LLNA and human reference data, as well as with other NAMs including DPRA, KeratinoSens™, and h-CLAT. LLNA outcomes are reported as positive (POS) or negative (NEG), with EC3 values provided when available. Human data are indicated when available. For each NAM, qualitative outcomes are reported as POS or NEG. SENS-IS results are expressed as a binary hazard call (sensitizer/non-sensitizer; S/NS) and as a potency category (Extreme, Strong, Moderate, Weak). “NA” indicates that no reliable or published data were available for the corresponding endpoint and therefore no comparison could be made.

**Table 2:** Comparison of SENS-IS hazard and potency outcomes obtained with EpiSkin™ and IS-SKIN reconstructed epidermis models for pre- and pro-haptens.

Name	CAS	Type	LLNA	EC3	SENS-IS S/NS EPISKIN	SENS-IS Potency Episkin	SENS-IS S/NS IS-SKIN	SENS-IS Potency IS-SKIN
Hydroquinone	123-31-9	pre-hapten	POS	0.11	POS	Strong	POS	Strong
Metol	55-55-0	pre-hapten	POS	0.8	POS	Strong	POS	Strong
2-aminophenol	95-55-6	pro-hapten	POS	0.4	POS	Strong	POS	Strong
Cinnamyl alcohol	104-54-1	pro-hapten	POS	21	POS	Weak	POS	Weak
4 allyl-anisol	140-67-0	pro-hapten	POS	20.2	POS	Moderate	POS	Moderate
Eugenol	97-53-0	pro-hapten	POS	13	POS	Moderate	POS	Moderate
Isoeugenol	97-54-1	pre-hapten	POS	1.2	POS	Strong	POS	Strong
Farnesol	4602-84-0	pro-hapten	POS	4.8	POS	Moderate	POS	Moderate
Geraniol	106-24-1	pro-hapten	POS	26	POS	Moderate	POS	Moderate
Benzo(a)pyrene	50-32-8	pro-hapten	POS	0.22	POS	Weak	POS	Weak
Propyl gallate	121-79-9	pre-hapten	POS	0.32	POS	Strong	POS	Strong
Lauryl gallate	1166-52-5	pre-hapten	POS	0.3	POS	Moderate	POS	Strong
Resorcinol	108-46-3	pre-hapten	POS	6.3	POS	Moderate	POS	Moderate
Abietic acid	514-10-3	pre-hapten	POS	11.0	POS	Weak	POS	Moderate
1-Naphthol	90-15-3	pro-hapten	POS	1.3	POS	Moderate	POS	Moderate
Limonene	5989-27-5	pre-hapten	POS	52	POS	Weak	POS	Weak

This table presents the SENS-IS hazard (sensitizer/non-sensitizer) and potency classifications obtained using the EpiSkin™ and IS-SKIN reconstructed human epidermis models for a subset of substances described in the literature as pre-haptens or pro-haptens. Results are compared with available Local Lymph Node Assay (LLNA) classifications and EC3 values. Potency categories were assigned according to the SENS-IS prediction model. The dataset includes substances used during the regulatory validation of the IS-SKIN model. Concordant hazard identification and comparable potency categorisation between EpiSkin™ and IS-SKIN were observed across the tested substances.

## Data Availability

The raw data supporting the conclusions of this article will be made available by the authors on request.

## Abbreviations

DMSO	Dimethyl Sulfoxide
SLS	Sodium Lauryl Sulfate
TNBS	2,4,6-Trinitrobenzene Sulfonic Acid
RhE	Reconstructed Human Epidermis
LLNA	Local Lymph Node Assay
